# Association of Hypertensive Disorders of Pregnancy and their Clinical Features with Peripartum Cardiomyopathy: A Systematic Review and Meta-analysis

**DOI:** 10.2174/011573403X329136250116050817

**Published:** 2025-02-13

**Authors:** Wilbert Huang, Siti Shofiah Syahruddin, Alexandra Aurelia Johansyah, Siti Saqinah Suriadiredja, Dhanny Primantara Johari Santoso, R.M. Sonny Sasotya, Muhammad Alamsyah Azis, Adhi Pribadi, Hawani Sasmaya Prameswari

**Affiliations:** 1 Medical School, Faculty of Medicine, Universitas Padjadjaran, Bandung, Indonesia;; 2 Department of Obstetrics and Gynecology, Faculty of Medicine, Universitas Padjadjaran, Bandung, Indonesia;; 3 Department of Cardiology and Vascular Medicine, Faculty of Medicine, Universitas Padjadjaran, Bandung, Indonesia

**Keywords:** Hypertensive disorders of pregnancy, preeclampsia, superimposed preeclampsia, gestational hypertension, chronic hypertension, peripartum cardiomyopathy

## Abstract

**Background:**

Peripartum Cardiomyopathy (PPCM) is a rare yet fatal cardiac disease associated with pregnancy. PPCM has been shown to have similar etiopathogenesis with hypertensive disorders of pregnancy (HDP). Hence, this study aims to study the association between HDP and the development of PPCM.

**Methods:**

Three databases (PubMed, Scopus, Cochrane Library) were searched and screened based on prespecified inclusion and exclusion criteria. Predictors of PPCM evaluated were HDP (preeclampsia, superimposed preeclampsia, chronic hypertension, and gestational hypertension) and its clinical features (severe preeclampsia, age, parity, serum creatinine, *etc*.). Data were analyzed using the random effects model of pooled odds ratios (ORs) with the Mantel Haenszel method, and publication bias was assessed with a funnel plot.

**Results:**

A total of 13 observational studies with 11,951 PPCM cases from 7 countries were identified. All types of HDP were associated with significantly increased odds of developing PPCM, and severe preeclampsia was associated with the highest OR of 13.33 (CI: 5.95 - 29.83, *p <* 0.01). Additionally, superimposed preeclampsia, chronic hypertension, preeclampsia, and lastly gestational hypertension were associated with increased odds of PPCM with OR 5.77, 4.73, 4.70, and 3.13, respectively. Other clinical features being statistically significant for PPCM development included advanced age > 35 years and multiple pregnancies (*p <* 0.05). No significant difference in creatinine level was found between PPCM and no PPCM group. No publication bias was found based on funnel plot assessment.

**Conclusion:**

HDP, especially severe preeclampsia, is associated with increased odds of PPCM development; hence, a low threshold for PPCM screening in this high-risk group is required.

## 1 INTRODUCTION

Peripartum Cardiomyopathy (PPCM) is a rare yet fatal idiopathic cardiac disease manifesting with heart failure symptoms developing in the last month of pregnancy up till otherwise healthy individuals and is associated with a substantial risk of cardiovascular morbidity cardiogenic shock, 5 months postpartum without any preexisting heart disease [1]. It is characterized by left ventricular systolic dysfunction with a dilated left ventricle chamber [2, 3]. It can develop in use of mechanical circulatory support and mortality caused by progressive heart failure, arrhythmia, and thromboembolism. The incidence of PPCM varies in each region, and this could be due to the different race domination that underlies the etiopathogenesis of PPCM [4-6]. Referral for genetic testing can also be considered for those at risk of developing heart failure due to positive family history of cardiomyopathy [7, 8]. Additionally, uncertainty in diagnosis is a common issue due to failure to rule out prior underlying cardiovascular disease, and delay in diagnosis of PPCM is common due to overlapping clinical manifestations of PPCM with normal late-term pregnancy [9]. Additionally, PPCM can also occur up till 5 months postpartum, which can lead PPCM to be unrecognized, hence delaying preventable complications. For this reason, understanding the etiopathogenesis of PPCM and identification of significant PPCM risk factors are important to screen high-risk pregnant women [10].

One of the proposed etiopathogenesis in PPCM includes the association of PPCM with preeclampsia, where there is a placental-derived proangiogenic/antiangiogenic disequilibrium that results in increased cleavage of prolactin into 16-kDa fragment that is proinflammatory, proapoptotic, antiangiogenic, and cardiotoxic [9, 11]. Besides that, the level of soluble Flt-1 and s-VEGFR-1, which are markers of antiangiogenic and proinflammatory response in preeclampsia, are also elevated in PPCM patients [12]. This association accounts for the interconnection of PPCM and hypertensive disorders of pregnancy (HDP). As of the day of literature searching, there is only one single-arm meta-analysis [13] studying the prevalence of preeclampsia in PPCM population, but it has not been able to show the increased risk/ odds of PPCM in preeclampsia and other hypertensive disorders of pregnancy (gestational hypertension, chronic hypertension, and superimposed preeclampsia). Moreover, other clinical features, such as severe preeclampsia, eclampsia, HELLP syndrome, and others, are also not studied. Hence, this systematic review and meta-analysis aimed to evaluate the association of hypertensive disorders of pregnancy and their clinical features with the incidence of PPCM.

## 2 METHODS

### 2.1 Search Strategy

A systematic search was performed on the main databases of PubMed, Scopus, and Cochrane Library up to January 2024, restricted only to the English language, but no date restriction was applied. The full search strategy is further described in Figs. (**S1**, **2**) and Tables (**S1**, **2**). This study has fulfilled the recommended 27-item checklist and a 4-phase flow diagram recommended from the Preferred Reporting Items for Systematic Reviews and Meta-Analyses (PRISMA) statements [14]. This review article has been registered in PROSPERO with the ID CRD42024507006.

### 2.2 Study Selection

With the basis of the therapeutic research question in this review, we selected observational and randomized controlled trial (when available) studies that included pregnant women with peripartum cardiomyopathy diagnosis. Studies were excluded when they reported only the incidence or prevalence of PPCM. Studies that evaluated the comparison of the PPCM group with other types of cardiomyopathy were also excluded. Additionally, case reports and case series were not included. Two investigators (WH and SSSy) independently evaluated the pre-selected studies, which complied with the inclusion criteria. Any disagreements were resolved through discussion with the other author (AAJ).

### 2.3 Risk of Bias Assessment

Observational studies included in this study each underwent a series of risk-of-bias assessments using the Revised Cochrane risk-of-bias tool for observational study protocol (ROBINS-I). The assessment for observational studies included the evaluation of study bias in the following domains: confounding variables, selection of participants, classification of interventions, deviations from intended interventions, missing data, measurement of outcomes, and selection of reported results. All components were considered in the overall risk of bias judgement. The series of risk assessments was performed independently by two reviewers (AAJ and SSSy) with disagreement resolved by consensus.

### 2.4 Data Extraction

Data from a total of 13 observational studies were independently retrieved and recorded by two reviewers (WH and SSSy). A third reviewer (SSS) evaluated all the collected data for any inconsistency, and all authors came to a consensus to discuss any existing inconsistency. From all of the selected studies, the data that were extracted included authors, name of registry, study design, year of publication, location of study, race of population, sample size, population included, age, gestational week, parity, hypertension disorders of pregnancy and their features, and PPCM definition.

### 2.5 Predictors and Outcome Measures

Outcome measures being analyzed in this study included the incidence of peripartum cardiomyopathy. Peripartum cardiomyopathy is defined as the onset of heart failure symptoms with no other cause in the last month of pregnancy or within 5 months postpartum with evidence of left ventricular systolic dysfunction as well as dilated left ventricle based on echocardiography findings.

Predictors analyzed in this study included hypertensive disorders of pregnancy and their clinical features. Conditions included in the group of hypertensive disorders of pregnancy were preeclampsia, superimposed preeclampsia, gestational hypertension, and chronic hypertension. Additional features analyzed in this study included severe preeclampsia, eclampsia, HELLP syndrome, and creatinine levels. Severe preeclampsia is defined as preeclampsia features with an addition of one or more of the following: severe hypertension (> 160/ 110 mmHg), severe proteinuria, signs of organ failure (elevated creatinine level or oliguria, hepatic dysfunction), new onset headache or visual disturbance, HELLP syndrome, and eclampsia [15].

### 2.6 Statistical Analysis

All aspects of this meta-analysis have been conducted according to the PRISMA guideline. We used Review Manager (RevMan) version 5.4 to conduct the statistical analysis. The odds ratio described in each study was pooled using the generic invariance method with a random effect model. Multivariable logistic regression result of odds ratio was chosen as the initial choice for analysis whenever possible. Studies that reported event rates were converted to odds ratio using the RevMan calculator. Additionally, mean difference or standardized mean difference was calculated for continuous outcomes when predictors were available. Subgroup difference was calculated with chi-square difference. The confidence interval of 95% was used, and statistical significance was determined when the p-value was below 0.05. Heterogeneity was assessed by I^2^ with an interpretation of I^2^ < 40% as not important heterogeneity, 30 - 60% as moderate heterogeneity, 50 - 90% as substantial heterogeneity, and 75% - 100% as considerable heterogeneity. Publication bias was assessed by funnel plot, and sensitivity and subgroup analyses were conducted based on several variables that might confound results accordingly. The GRADE assessment tool was used to assess the certainty of evidence presented in this study [16, 17].

## 3 RESULTS

### 3.1 Study Selection and Characteristics

**Fig. (1):**
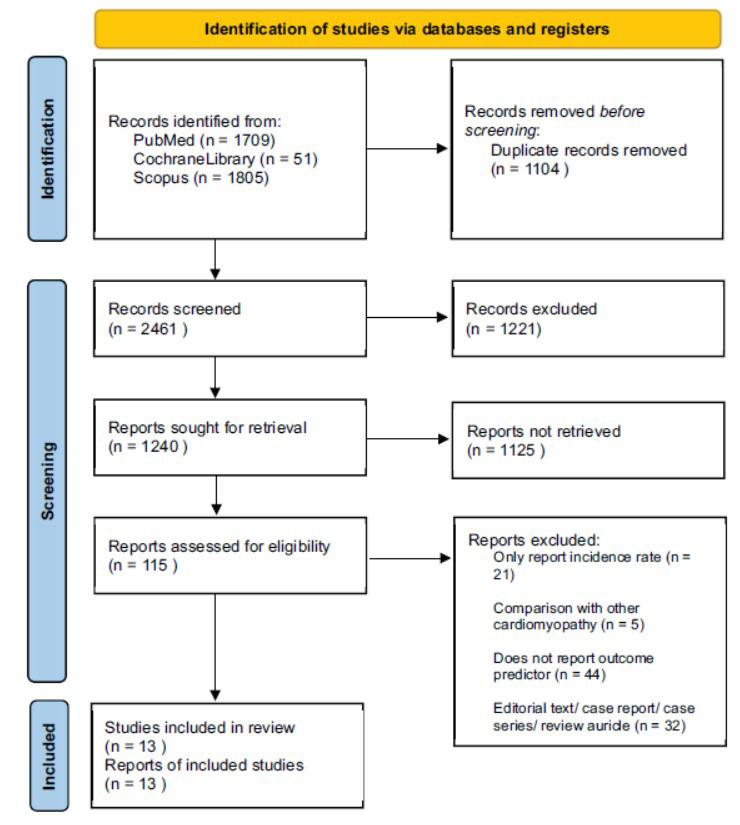
PRSIMA flow chart diagram.

Initial searching results from three databases (PubMed, Scopus, CochraneLibrary) resulted in a total of 3565 identified studies where 1104 duplicates were removed, and after screening for title, abstract, and full-text articles, eventually, 13 observational studies [8-20] were retrieved and included in this study (Fig. **1**). A total of 11, 951 PPCM cases from 7 countries were included, in which cases were identified from 1993 - 2020. The majority of the studies included only pregnant women aged > 15 years old and were followed up for 1 week up to 5 months postpartum. Ten studies recorded that 15% of the total population was above 35 years old, six studies recorded that 53.3% of them were primiparity, four studies recorded that 1.9% of them were grand multiparity, and eleven studies recorded that 1.7% of them had multiple pregnancies (Table **1**).

### 3.2 Risk of Bias Assessment

Risk of bias assessment with the ROBINS-I tool found that 5 studies were regarded to have some concerns of bias while the remaining were assessed as having low risk of bias. Bias due to confounding was found in 4 out of 13 studies, and bias in participant selection was found in 2 out of 14 studies. One study was evaluated as having some concern of bias due to missing data (Table **1**).

### 3.3 Data Synthesis

Eleven studies reported the association between preeclampsia and the incidence of PPCM, where preeclampsia was present in 13.5% of all PPCM cases. It was shown that pregnant women with preeclampsia had statistically significant 4.70 times the odds of developing PPCM after sensitivity analysis of only multivariate analysis studies (CI: 3.05 - 7.256, *p <* 0.01, I^2^ = 97%). In the group without PPCM, 1.1 - 3.0% of them had preeclampsia. Five studies reported the association of gestational hypertension with PPCM, where it was present in 5.7% of all PPCM cases, and gestational hypertension resulted in a significant pooled odds ratio of 3.13 for developing PPCM (CI: 1.87 - 5.25, *p <* 0.01, I^2^ = 93%). In the group without PPCM, 5.9 - 6.2% of them had gestational hypertension. Six studies reported the association of chronic hypertension and PPCM, where it was present in 11.2% of all PPCM cases and resulted in a significant pooled odds ratio of 4.73 (CI: 3.33 - 6.73, *p <* 0.01, I^2^ = 86%). In the group without PPCM, 2.4 - 6.4% of them had chronic hypertension.

**Fig. (2):**
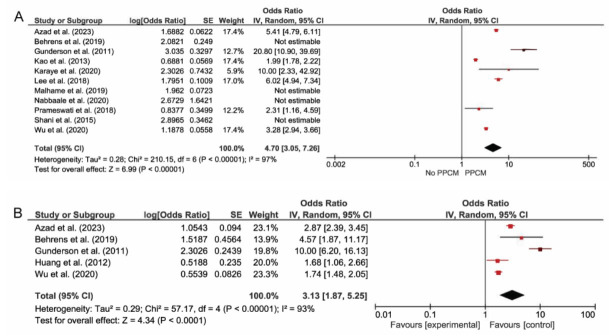
Random effects pooled odds ratio of (**A**) preeclampsia (**B**) gestational hypertension with the development of PPCM. Both preeclampsia and gestational hypertension is associated with an increased odds of PPCM development (*p <* 0.01).

**Fig. (3):**
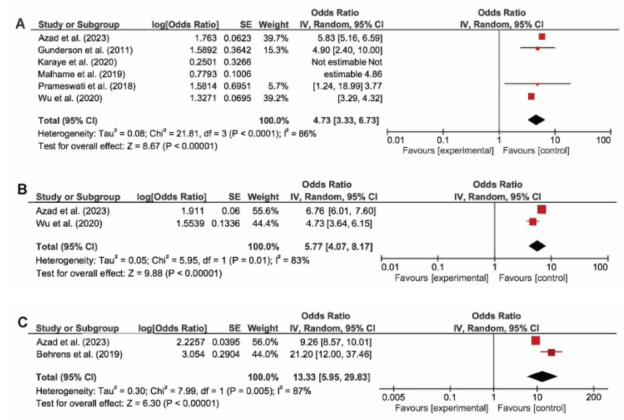
Random effects pooled odds ratio of (**A**) chronic hypertension (**B**) superimposed preeclampsia (**C**) severe preeclampsia with the development of PPCM. Severe preeclampsia results in the highest odds ratio compared to other hypertensive disorders of pregnancy.

Only two studies reported the association of superimposed preeclampsia and PPCM, and it resulted in a significant pooled odds ratio of 5.77 (CI: 4.07 - 8.17, *p <* 0.01, I^2^ = 83%). All four types of hypertensive disorders of pregnancy resulted in statistically significant increased odds of developing PPCM; however, the results had a substantial heterogeneity. Sensitivity analysis done by leaving one study out at a time did not result in a significant change of heterogeneity (Figs. **2** and **3**). Additional subgroup analysis comparing odds of preeclampsia and odds ratio showed that the pooled odds ratio of the 2 predictors were not statistically different (*p* 0.98).

Two studies reported the association of severe preeclampsia and PPCM, where it was present in 20.6% of all PPCM cases and resulted in the highest pooled odds ratio of 13.33 (CI: 5.95 - 29.83, *p <* 0.01, I^2^ = 87%) (Fig. **3**). Only one study reported an association between eclampsia and the incidence of PPCM, where eclampsia was present in 0.1% of the no PPCM group and 2.1% in the PPCM group. Besides that, one study reported an association between HELLP syndrome and PPCM, where HELLP syndrome was present in 0.4% of the no PPCM group and 9.1% in the PPCM group.

Additional clinical features identified and analyzed in this study included age > 35 years old, primiparity, grand multiparity, and multiple pregnancies. Only age > 35 years old was associated with a significant increase in the incidence of PPCM with an increasing odd of 1.90 (CI: 1.73 - 2.08, *p =* 0.015, I^2^ = 32%). When a low sample size study of < 1000 was excluded in this analysis, grand multiparity was associated with an increasing odd of PPCM with OR 3.54 (CI: 1.88 - 6.67, *p =* 0.01, I2 = 84%). Pooled serum creatinine levels between PPCM and no PPCM group from 2 studies did not result in a significant standardized mean difference between the groups (*p =* 0.62) (Figs. **1** and **2**).

Publication bias assessed by the funnel plot did not show any asymmetrical figure; hence, there was no significant publication bias in each of the predictors analyzed (Fig. **3**).

GRADE assessment for evidence presented in each prognostic factor of outcome can be found in Fig. (**4**). All the prognostic factors except for severe preeclampsia had a moderate level of certainty, while the latter had a low level of certainty.

## 4 DISCUSSION

This study shows that severe preeclampsia is associated with the highest odds of developing PPCM, which is followed by superimposed preeclampsia, chronic hypertension, preeclampsia, and gestational hypertension. However, it is also shown that hypertension disorders of pregnancy only occur in a small proportion of women with PPCM. Additional clinical features associated with PPCM include advanced age > 35 years old, multiple pregnancies, and grand multiparity.

The overall substantial heterogeneity in this study may reflect the role of genetic predisposition of different races in the etiopathogenesis of PPCM [3]. This is also complicated by different definitions of PPCM used in each study, which may result in under or overreporting the incidence of PPCM [18]. However, in this study, we are not able to describe further the racial difference between studies due to limited data presented in the included studies; hence, no subgroup analysis can be conducted.

In this study, the small proportion of hypertension disorders of pregnancy in PPCM patients supports the 2 hit theory of PPCM, where the first hit is attributed to the antiangiogenic and cardiotoxic properties of the 16-kDa fragment, sFlt-1, enzyme cathepsin D, and other mediators associated with hypertensive disorders of pregnancy [19, 20]. While the second hit is the underlying factors that increase the susceptibility of certain pregnant women to develop PPCM. These factors include reduced cardiac proangiogenic defenses, myocarditis, genetic predisposition, and viral infection [2, 12]. The 2-hit theory is further supported by the fact that 18 - 28% of apparently healthy nulliparous women had left ventricular diastolic dysfunction at term due to increased hemodynamic load [21]. When these women are predisposed to the second hit factor, the stress of pregnancy, it may hasten the development of PPCM. The second hit phenomenon has been difficult to be identified and remains a knowledge gap in understanding peripartum cardiomyopathy.

We found that severe preeclampsia is associated with the highest odds of developing PPCM, and this finding is in line with the second hit theory, where there is a significant cardiac maladaptation that includes severe myocardial remodeling with worse left ventricular global systolic and diastolic function in severe preeclampsia [22]. Additionally, severe preeclampsia is also associated with a greater risk of developing major adverse cardiovascular events within a year of PPCM diagnosis [9, 23].

Additionally, longer exposure to cardiac insults, such as in chronic hypertension, is seen to have a higher risk of developing PPCM when compared to gestational hypertension, which occurs only after the 20^th^ week of gestation [24]. Chronic exposure to cardiac insults due to a prolonged hypertension state could result in vascular inflammation, which will provoke vascular inflammation in the placenta, disrupt the angiogenic balance, and eventually result in a worse clinical disease [25]. In line with this finding is the higher odds of outcome in the superimposed preeclampsia group when compared to preeclampsia. Interestingly, we found that chronic hypertension resulted in a slightly higher pooled odds ratio compared to preeclampsia, but this finding is not statistically significant.

Several other identified risk factors for PPCM, including advanced age > 35 years old, multiple pregnancy, and grand multiparity, further support the association of hypertensive disorders of pregnancy (particularly preeclampsia) and the development of PPCM due to the shared risk factors between them [26, 27].

Nonetheless, in this study, we were not able to identify the second hit factors associated with PPCM development and were only able to show the increasing odds of PPCM in hypertensive disorders of pregnancy. Hence, until certain second-hit risk factors can be successfully identified, a low threshold for performing echocardiography screening within high-risk groups (mainly severe preeclampsia) is important to not miss out on any PPCM case. Additionally, evaluation with biomarkers known to predict preeclampsia can also be used to stratify risk in PPCM cases because of their interlinking relationship [28-30]. Additionally, echocardiography parameters from speckle tracking analysis, such as left atrial reservoir, can also be used to predict HDP [31].

## 5 LIMITATIONS AND RECOMMENDATIONS

This study has several limitations that could be further addressed in future studies. First, included studies were not able to distinctly discern antepartum and postpartum PPCM cases in which the period when PPCM cases develop can aid in increasing awareness in high-risk populations. Second, this study was also not able to identify other important clinical features of hypertensive disorders of pregnancy, such as blood pressure, proteinuria, thrombocyte number, and liver enzyme level, due to limited presented data from the included studies. Third, our study was also not able to statistically compare the odds ratio of one PPCM predictor to the other hence, the conclusion drawn is only done narratively. Lastly, we were also unable to determine the LV dysfunction severity during the PPCM diagnosis due to the limited data presented in the included studies. We recommend that future studies should be conducted to study the outcome of patients with HDPs who develop PPCM. Long-term cohort studies in this population will reveal the prognosis of these patients and will be impactful for clinicians in monitoring PPCM patients.

## CONCLUSION

Hypertensive disorders of pregnancy (especially severe preeclampsia) are associated with increasing odds of PPCM development; hence, a lower threshold for PPCM screening should be considered in this high-risk group. PPCM has also been found to share similar risk factors with preeclampsia, which further substantiates their correlation.

## Figures and Tables

**Fig. (4):**
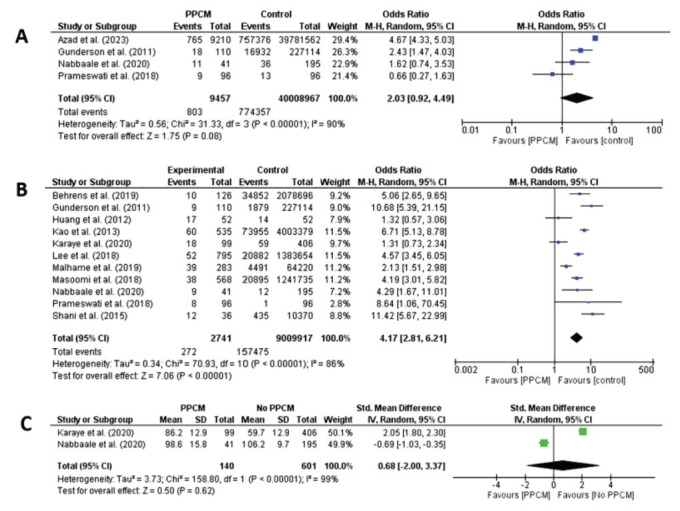
(**A-C**) GRADE assessment of each prognostic factor associated with odds of PPCM incidence. All prognostic factors except for severe preeclampsia have a moderate level of certainty.

**Table 1:** Characteristics of included studies.

**Author**		**Study Time Period**	**Country**	**Race**	**Sample Size (Total)**	**Sample Size (PPCM)**	**Population**	**Age (PPCM) ± SD/ (IQR)**	**Age > 35 (PPCM) n (%)**	**Primipara (PPCM) n (%)**	**Grand Multipara (PPCM) n (%)**	**Multiple Pregnancy (PPCM) n (%)**		**PPCM Definition**	**PPCM (Antepartum/ Postpartum)**			**Follow up Period**
Azad (2023)		2010 - 2020	USA	Not specified (62% US residents)	39,790,772	9,210	Pregnant women aged 15 - 54 years (Discharge occurred from 1 January through 31 July for each year)	-	2391 (25.9)	-	765 (8.3)		-	Diagnoses of peripartum cardiomyopathy were identified based on ICD-9-CM code 674.5x and ICD-10-CM code O90.3.	Both	5 months		
Behrens (2019)		1978 - 2012	Denmark	Not specified	2,078, 822	126	All pregnancies in Denmark ending in live birth or stillbirth between 1978 and 2012 Excluded: 1. Pregnancies of <20 weeks’ duration and patient with any cardiovascular disease and diabetes up to 30 days before their first delivery 2. Pregnancies occuring after cardiomyopathy, heart failure, or ischemic heart disease	-	37 (29.4)	57 (45.2)	-		10 (7.9)	Cardiomyopathy registered in the National Patient Register or Causes of Death Register (ICD-8: 425.99; ICD-10: I42.0-43.8, O90.3) in the peripartum period, defined as the 6-month period beginning 1 month before delivery and ending 5 months postpartum, without registration of cardiomyopathy prior to the pregnancy	Both	30 days before to 7 days after delivery		
Gunders-on (2011)		1995 - 2004	USA	Non- hispanic white (41.0/ 34.5) Non hispanic African american (9.3 / 29.1) Hispanic (26.7/ 8.2) Chinese (4.0/ 1.8) Filipina (6.9/ 14.5) Other Asian or Pacific Islander (4.6/ 3.6) Other race or ethnicity (7.6/ 8.2)	227, 224	110	All live births that occurred at any Kaiser Permanente of Northern California hospital between January 1, 1995, and December 31, 2004 Excluded: 1. Mother-newborn pairs who had inaccurate medical record numbers, no accessible delivery records, missing data on maternal age or length of gestation, or for whom we could not find a match in the neonatal birth certificate files 2. Women who had a diagnosis of heart failure or valvular heart disease before the selected pregnancy in the study period	-	38 (34.5)	43 (39.1)	18 (16.4)		9 (8.2)	Onset of clinical heart failure with no identifiable cause in the last month of pregnancy or within 5 months after delivery in the absence of heart disease before the last month of pregnancy with echocardiographic evidence of left ventricular systolic dysfunction such as reduced fractional shortening or ejection fraction as well as a dilated left ventricle (National Heart, Lung and Blood Institute criteria)	Both	Up to 36 months postdelivery		
Huang (2012)	2007 - 2009		China	Not specified (Shandong, China residents)	104	52	Pregnant patients admitted to Liaocheng People’s Hospital from May 2007 to May 2009	29.1 ± 6.3	-	-	-	17 (32.7)		Diagnosis fo PPCM was suggested when patients presented with heart failure symptoms in late pregnancy or within the first five months postpartum - Heart failure symptoms were verified by transthoracic echocardiographic examination, which showed a left ventricular EF of less than 45% - Other causes for heart failure and cardiomyopathy, such as pre-existing hypertension, coronary heart disease, heart valve disease, congenital heart disease, alcoholic cardiomyopathy and tachycardia-induced heart disease, had to be excluded	Both			14-34 days
Kao (2013)	2003 - 2007		USA	Caucasian (36/ 32.3%) African american (8.1/ 19.6) Hispanic (29.6/ 23.0) Other (10.7/ 6.9) Not available (15.6/ 18.1)	4,003,914	535	39,829,857 hospital records from 620 hospitals from January 2003 through December 2007 with specified outcome of delivery	-	30 (5.6)	-	-	60 (11.2)		A record was classified as indicating PPCM if it included any of the ICD-9 CM codes 674.50 to 55	Antepartum			-
Karaye (2020)	2017 - 2018		Nigeria	Patients belonged to 29 different ethnic groups, 311 (76.6%) of them were of Hausa-Fulani ethnicity, 5.4% were Kanuris, and the Nupes and Yorubas each represented 3.9% of the cohort.	505	99	406 patients from 22 centres in Nigeria and compared with 99 controls from seven of the centres, between June 2017 and March 2018	28.6 ± 7.2	140 (34.5)	-	-	59(14.5)		An idiopathic cardiomyopathy presenting with HF secondary to LV systolic dysfunction (SD) towards the end of pregnancy or in the months following delivery, where no other cause of HF is found (a diagnosis of exclusion).	Both			Not specified
Lee (2018)		2009 - 2013	Korea	Not specified (Korean residents)	1,384, 449	795	Patients who fulfilled predefined diagnostic criteria for PPCM (International Classification of Diseases, Tenth Revision, Clinical Modification codes) from January 1, 2010, to December 31, 2012 Excluded: 1. Subjects who already had heart failure related International Classification of Diseases, Tenth Revision, Clinical Modification codes at least 1 year before delivery	32.1±4.3	228 (28.7)	439 (55.2)	-	52 (6.5)		An idiopathic pregnancy-associated heart failure (HF) that develops toward the end of pregnancy or in the following months after delivery in a woman without previously known structural heart disease (Heart Failure Association of the European Society of Cardiology Working Group on PPCM)	Both		1 month antepartum to 5 months postpartum	
Malhame (2019)		2010 - 2014	USA	Not specified	64503	283	Women aged 15-55 years with obstetric deliveries between April 1st, 2011 and June 30th, 2014 and insurance coverage for at least 6 months prior to the estimated date of conception.	31.8	109 (38.5)	-	-	39 (13.8)		Diagnostic code for PPCM and a procedure code for cardiac echocardiography between the last month of gestation and the first 5 months postpartum were considered as having PPCM	Both		5 months postpartum	
Masoomi (2013)		2013	USA	Not specified (62% US residents)	1,242, 303	568	Women aged 15 - 54 years (Discharge occurred from 1 January through 31 July for each year)	30.0 (29.34-30.57)	126 (22.2)	-	-	38 (6.7)		Confirmed reduced left ventricular systolic function and exclusion of pre-existing cardiac conditions and other etiologies of heart failure	Both		Immediate post delivery period and up to 3 months post-delivery.	
Nabbaale (2020)		2018 - 2019	Uganda	Black African	236	41	Any women >= 18 years presenting with symptoms of congestive cardiac failure that developed in the last month of pregnancy or during the first five months postpartum, without any identifiable cause for heart failure like coronary artery disease, pre-existing hypertension, rheumatic heart disease and congenital heart disease	35 (27-39)	18(46.1)	9 (21.9)	11 (26.8)		9 (21.9)	An idiopathic cardiomyopathy presenting with heart failure secondary to LV systolic dysfunction (LVEF <45%) towards the end of pregnancy or in the months following delivery, if no other cause of heart failure is found (European Society of Cardiology)	both	6 months		
Prames-wari (2018)		2011 - 2014	Indonesia	Not specified	192	96	Newly diagnosed PPCM and the control group is defined as clinically normal parturietn between last trimester of pregnancty until 5 months after delivery with no sign and symptoms related to PPCM	30.5 ± 13	29 (30.2)	33 (34.4)	9 (9.4)		8 (8.3)	An idiopathic cardiomyopathy with symptoms and signs of heart failure, secondary to disorders of ventricular systolic function, in late pregnancy or postpartum, where no other cause of heart failure is found	both	5 months postpartum		
Shani (2015)		1993 - 2010	Israel	2.8% African, 97.2% Caucasian (PPCM)	10406	36	All women who gave birth at their medical center during the calendar year 2007	33.5 ± 6	-	23 (63.9)	-		12 (33.3)	The onset of cardiac failure with no identifiable cause in the last month of pregnancy or within 5 months after delivery, in the absence of known heart disease	both	0-120 days following delivery		
Wu (2020)		2004 - 2014	USA	-	44276975	-	Inpatient pregnant women from 2004 - 2014 in the database	-	-	-	-		-	-	-	-		

## Data Availability

The data and supportive information are available within the article.

## LIST OF ABBREVIATIONS

PPCM: Peripartum Cardiomyopathy
HDP: Hypertensive Disorders of Pregnancy
PRISMA: Preferred Reporting Items for Systematic Reviews and Meta-Analyses

## STANDARDS OF REPORTING

PRISMA guidelines and methodology were followed.

## SUPPLEMENTARY MATERIAL

PRISMA checklist and supplementary material are available on the publisher’s website along with the published article.
