# Supplementary material for: Association of Hypertensive Disorders of Pregnancy and their Clinical Features with Peripartum Cardiomyopathy: A Systematic Review and Meta-analysis
Source: Curr Cardiol Rev. 2025 Feb 13;21(6):e1573403X329136. doi: 10.2174/011573403X329136250116050817 (PMC12676040; doi:10.2174/011573403X329136250116050817)
Supplement: Supplementary file 1 [file CCR-21-6-E1573403X329136_SD1.pdf]

Supplementary Material

Association of Hypertensive Disorders of Pregnancy and their Clinical Features with Peripartum Cardiomyopathy: A Systematic Review and Meta-analysis

Wilbert Huang[1,*], Siti Shofiah Syahruddin[1], Alexandra Aurelia Johansyah[1], Siti Saqinah Suriadiredja[1], Dhanny Primantara Johari Santoso[2], RM Sonny Sasotya[2], Muhammad Alamsyah Azis[2], Adhi Pribadi[2] and Hawani Sasmaya Prameswari[3]

[1]*Medical School, Faculty of Medicine, Universitas Padjadjaran, Bandung, Indonesia;* [2]*Department of Obstetrics and Gynecology, Faculty of Medicine, Universitas Padjadjaran, Bandung, Indonesia;* [3]*Department of Cardiology and Vascular Medicine, Faculty of Medicine, Universitas Padjadjaran, Bandung, Indonesia*

Section and Topic	Item #	Checklist item	Location where item is reported
**TITLE**			
Title	1	Identify the report as a systematic review.	Title
**ABSTRACT**			
Abstract	2	See the PRISMA 2020 for Abstracts checklist.	Done
**INTRODUCTION**			
Rationale	3	Describe the rationale for the review in the context of existing knowledge.	Introduction
Objectives	4	Provide an explicit statement of the objective(s) or question(s) the review addresses.	Introduction
**METHODS**			
Eligibility criteria	5	Specify the inclusion and exclusion criteria for the review and how studies were grouped for the syntheses.	Methods
Information sources	6	Specify all databases, registers, websites, organisations, reference lists and other sources searched or consulted to identify studies. Specify the date when each source was last searched or consulted.	Methods
Search strategy	7	Present the full search strategies for all databases, registers and websites, including any filters and limits used.	Methods
Selection process	8	Specify the methods used to decide whether a study met the inclusion criteria of the review, including how many reviewers screened each record and each report retrieved, whether they worked independently, and if applicable, details of automation tools used in the process.	Methods
Data collection process	9	Specify the methods used to collect data from reports, including how many reviewers collected data from each report, whether they worked independently, any processes for obtaining or confirming data from study investigators, and if applicable, details of automation tools used in the process.	Methods
Data items	10a	List and define all outcomes for which data were sought. Specify whether all results that were compatible with each outcome domain in each study were sought (e.g. for all measures, time points, analyses), and if not, the methods used to decide which results to collect.	Methods
	10b	List and define all other variables for which data were sought (e.g. participant and intervention characteristics, funding sources). Describe any assumptions made about any missing or unclear information.	Methods
Study risk of bias assessment	11	Specify the methods used to assess risk of bias in the included studies, including details of the tool(s) used, how many reviewers assessed each study and whether they worked independently, and if applicable, details of automation tools used in the process.	Methods
Effect measures	12	Specify for each outcome the effect measure(s) (e.g. risk ratio, mean difference) used in the synthesis or presentation of results.	Methods
Synthesis methods	13a	Describe the processes used to decide which studies were eligible for each synthesis (e.g. tabulating the study intervention characteristics and comparing against the planned groups for each synthesis (item #5)).	Methods
	13b	Describe any methods required to prepare the data for presentation or synthesis, such as handling of missing summary statistics, or data conversions.	Methods
	13c	Describe any methods used to tabulate or visually display results of individual studies and syntheses.	Methods

Section and Topic	Item #	Checklist item	Location where item is reported
	13d	Describe any methods used to synthesize results and provide a rationale for the choice(s). If meta-analysis was performed, describe the model(s), method(s) to identify the presence and extent of statistical heterogeneity, and software package(s) used.	Methods
	13e	Describe any methods used to explore possible causes of heterogeneity among study results (e.g. subgroup analysis, meta-regression).	Methods
	13f	Describe any sensitivity analyses conducted to assess robustness of the synthesized results.	Methods
Reporting bias assessment	14	Describe any methods used to assess risk of bias due to missing results in a synthesis (arising from reporting biases).	Methods
Certainty assessment	15	Describe any methods used to assess certainty (or confidence) in the body of evidence for an outcome.	-
**RESULTS**			
Study selection	16a	Describe the results of the search and selection process, from the number of records identified in the search to the number of studies included in the review, ideally using a flow diagram.	Results
	16b	Cite studies that might appear to meet the inclusion criteria, but which were excluded, and explain why they were excluded.	Results
Study characteristics	17	Cite each included study and present its characteristics.	Results
Risk of bias in studies	18	Present assessments of risk of bias for each included study.	Results
Results of individual studies	19	For all outcomes, present, for each study: (a) summary statistics for each group (where appropriate) and (b) an effect estimate and its precision (e.g. confidence/credible interval), ideally using structured tables or plots.	Results
Results of syntheses	20a	For each synthesis, briefly summarise the characteristics and risk of bias among contributing studies.	Results
	20b	Present results of all statistical syntheses conducted. If meta-analysis was done, present for each the summary estimate and its precision (e.g. confidence/credible interval) and measures of statistical heterogeneity. If comparing groups, describe the direction of the effect.	Results
	20c	Present results of all investigations of possible causes of heterogeneity among study results.	Results
	20d	Present results of all sensitivity analyses conducted to assess the robustness of the synthesized results.	Results
Reporting biases	21	Present assessments of risk of bias due to missing results (arising from reporting biases) for each synthesis assessed.	Results
Certainty of evidence	22	Present assessments of certainty (or confidence) in the body of evidence for each outcome assessed.	-
**DISCUSSION**			
Discussion	23a	Provide a general interpretation of the results in the context of other evidence.	Discussion
	23b	Discuss any limitations of the evidence included in the review.	Discussion
	23c	Discuss any limitations of the review processes used.	Discussion
	23d	Discuss implications of the results for practice, policy, and future research.	Discussion
**OTHER INFORMATION**			
Registration and protocol	24a	Provide registration information for the review, including register name and registration number, or state that the review was not registered.	Methods
	24b	Indicate where the review protocol can be accessed, or state that a protocol was not prepared.	Methods
	24c	Describe and explain any amendments to information provided at registration or in the protocol.	-
Support	25	Describe sources of financial or non-financial support for the review, and the role of the funders or sponsors in the review.	Done
Competing interests	26	Declare any competing interests of review authors.	Done
Availability of data, code and other materials	27	Report which of the following are publicly available and where they can be found: template data collection forms; data extracted from included studies; data used for all analyses; analytic code; any other materials used in the review.	Done

From: Page MJ, McKenzie JE, Bossuyt PM, Boutron I, Hoffmann TC, Mulrow CD, *et al*. The PRISMA 2020 statement: an updated guideline for reporting systematic reviews. BMJ 2021;372:n71. doi: 10.1136/bmj.n71

For more information, visit: http://www.prisma-statement.org/

SUPPLEMENTARY

PubMed, Scopus, CochraneLibrary:

(“peripartum cardiomyopathy” OR “pregnancy associated cardiomyopathy” OR “pregnancy- associated cardiomyopathy” OR “postpartum cardiomyopathy” OR “prepartum cardiomyopathy” OR “peripartum heart failure” OR “PPCM”)

Table S1. Risk of bias assessment with ROBINS-I tool.

Study	Bias Due to Confounding	Bias Arising from Measurement of the Exposure	Bias in Selection of Participants	Bias due to Post-Exposure Interventions	Bias due to Missing Data	Bias in Measurement of Outcomes	Bias in Selection of the Reported Result	Overall Risk of Bias Judgement
Azad *et al*, 2023	Some concerns	Low	Low	Low	Low	Low	Low	Low
Behrens *et al*, 2019	Low	Low	Low	Low	Low	Low	Low	Low
Gunderson *et al*, 2011	Low	Low	Low	Low	Low	Low	Low	Low
Huang *et al*, 2012	Low	Low	Some concerns	Low	Low	Low	Low	Some concerns
Kao *et al*, 2013	Low	Low	Some concerns	Low	Some concerns	Low	Low	Some concerns
Karaye *et al*, 2020	Low	Low	Low	Low	Low	Low	Low	Low
Lee *et al*, 2018	Low	Low	Low	Low	Low	Low	Low	Low
Malhame *et al*, 2019	Some concerns	Low	Low	Low	Low	Low	Low	Some concerns
Masoomi *et al*, 2018	Some concerns	Low	Low	Low	Low	Low	Low	Some concerns
Nabbaale *et al*, 2020	Some concerns	Low	Low	Low	Low	Low	Low	Some concerns
Prameswari *et al*, 2018	Low	Low	Low	Low	Low	Low	Low	Low
Shani *et al*, 2015	Low	Low	Low	Low	Low	Low	Low	Low
Wu *et al*, 2020	Low	Low	Low	Low	Low	Low	Low	Low

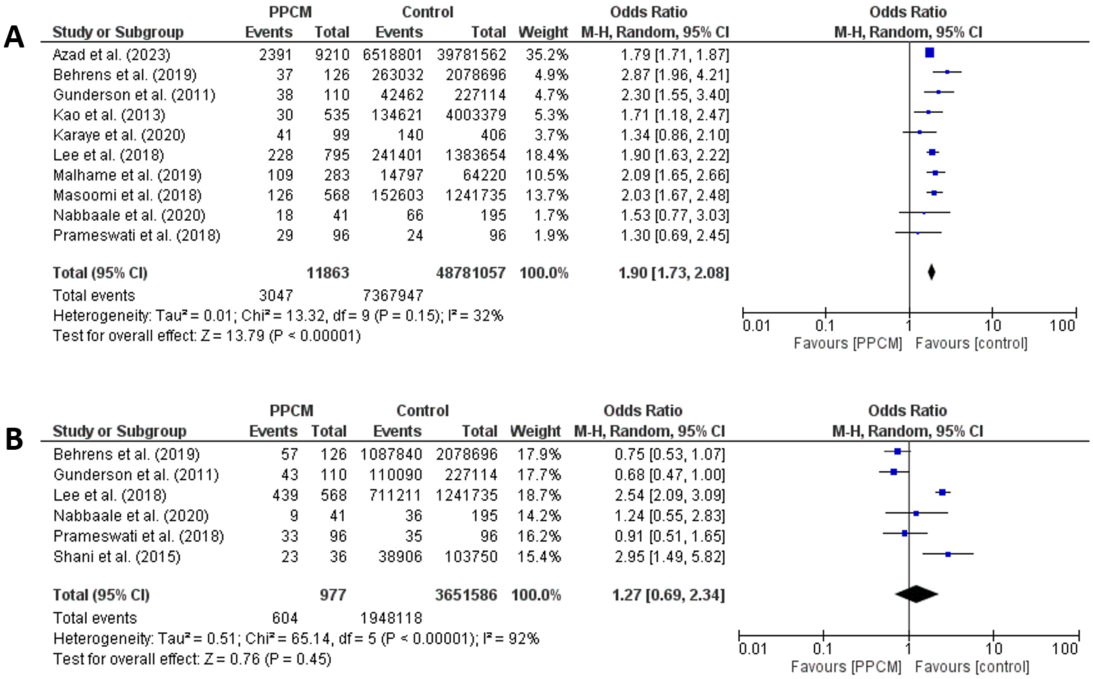

Fig. (S1). Random effects pooled odds ratio of (**A**) advanced age > 35 years (**B**) primiparity with the development of PPCM. Advanced age but not primiparity is significantly associated with the development of PPCM.

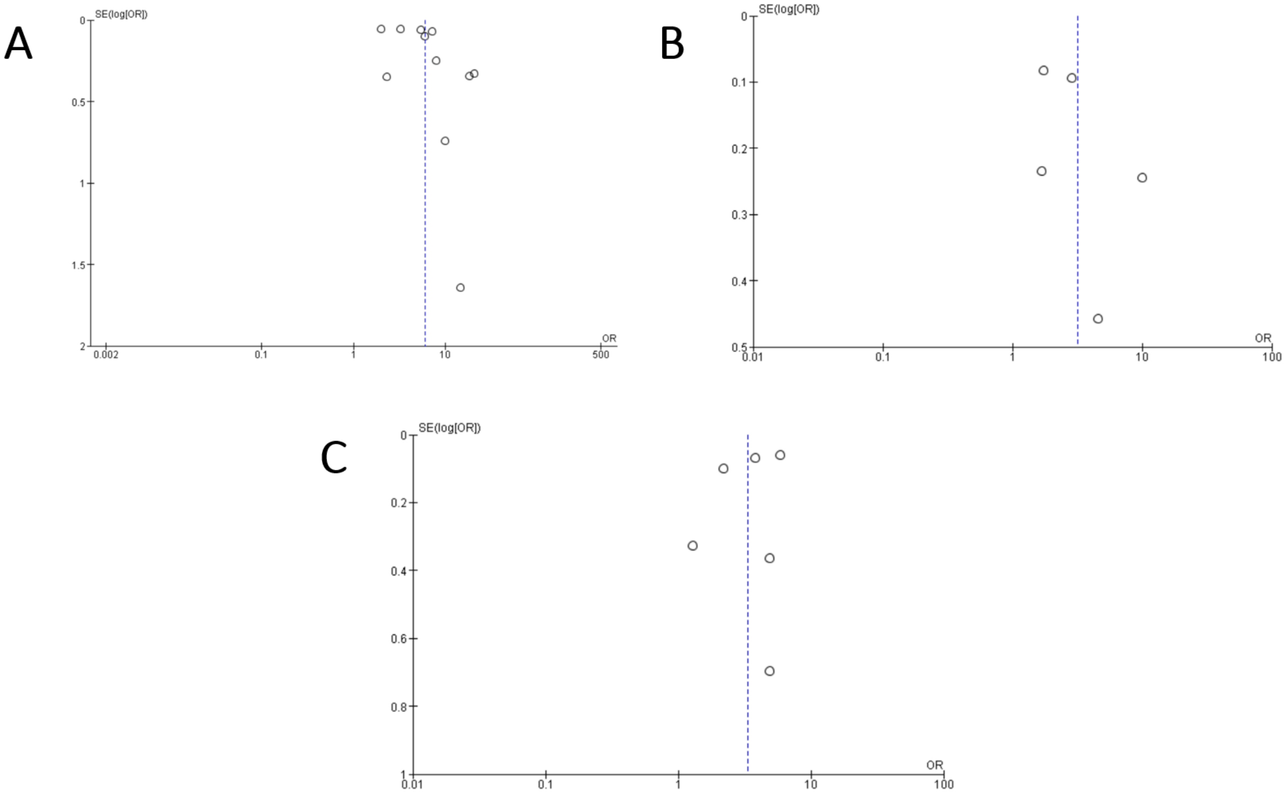

Fig. (S2). Funnel plot assessment on predictors of (**A**) preeclampsia (**B**) gestational hypertension (**C**) chronic hypertension. No asymmetries are observed hence publication bias is not present.

Table S2. GRADE assessment of each prognostic factors associated with odds of PPCM incidence. All prognostic factors except for severe preeclampsia has moderate level of certainty.

№ of studies	Certainty assessment						Effect			Certainty	Importance
	Study design	Risk of bias	Inconsistency	Indirectness	Imprecision	Other considerations	№ of events	№ of individuals	Rate (95% CI)		
**Preeclampsia**											
11	non-randomised studies	serious	not serious	not serious	serious[a]	all plausible residual confounding would suggest spurious effect, while no effect was observed				⊕⊕⊕○ Moderate	
**Gestational Hypertension**											
5	non-randomised studies	serious[a]	not serious	not serious	serious[a]	all plausible residual confounding would suggest spurious effect, while no effect was observed				⊕⊕⊕○ Moderate	
**Chronic Hypertension**											
4	non-randomised studies	serious	not serious	not serious	serious[a]	all plausible residual confounding would suggest spurious effect, while no effect was observed				⊕⊕⊕○ Moderate	
**Superimposed preeclampsia**											
2	non-randomised studies	serious	not serious	not serious	serious[a]	all plausible residual confounding would suggest spurious effect, while no effect was observed				⊕⊕⊕○ Moderate	
**Severe Preeclampsia**											
2	non-randomised studies	serious	serious[b]	not serious	serious[a]	all plausible residual confounding would suggest spurious effect, while no effect was observed				⊕⊕○○ Low	

Note: a. Not all studies use multivariate regression to adjust for confounding effects; b. Effect size of the study differs by alot.
